# Cu-Based Thermocompression Bonding and Cu/Dielectric Hybrid Bonding for Three-Dimensional Integrated Circuits (3D ICs) Application

**DOI:** 10.3390/nano13172490

**Published:** 2023-09-04

**Authors:** Yuan-Chiu Huang, Yu-Xian Lin, Chien-Kang Hsiung, Tzu-Heng Hung, Kuan-Neng Chen

**Affiliations:** 1 Institute of Electronics Engineering, National Yang Ming Chiao Tung University, Hsinchu 300, Taiwan; 2 International College of Semiconductor Technology, National Yang Ming Chiao Tung University, Hsinchu 300, Taiwan

**Keywords:** 3D ICs, advanced packaging, Cu-Cu bonding, hybrid bonding, low-temperature bonding

## Abstract

Advanced packaging technology has become more and more important in the semiconductor industry because of the benefits of higher I/O density compared to conventional soldering technology. In advanced packaging technology, copper–copper (Cu-Cu) bonding has become the preferred choice due to its excellent electrical and thermal properties. However, one of the major challenges of Cu-Cu bonding is the high thermal budget of the bonding process caused by Cu oxidation, which can result in wafer warpage and other back-end-of-line process issues in some cases. Thus, for specific applications, reducing the thermal budget and preventing Cu oxidation are important considerations in low-temperature hybrid bonding processes. This paper first reviews the advancements in low-temperature Cu-based bonding technologies for advanced packaging. Various low-temperature Cu-Cu bonding techniques such as surface pretreatment, surface activation, structure modification, and orientation control have been proposed and investigated. To overcome coplanarity issues of Cu pillars and insufficient gaps for filling, low-temperature Cu-Cu bonding used, but it is still challenging in fine-pitch applications. Therefore, low-temperature Cu/SiO_2_, Cu/SiCN, and Cu/polymer hybrid bonding have been developed for advanced packaging applications. Furthermore, we present a novel hybrid bonding scheme for metal/polymer interfaces that achieves good flatness and an excellent bonding interface without the need for the chemical mechanical polishing (CMP) process.

## 1. Introduction

Over the past few decades, the miniaturization of integrated circuits has been driven by the scaling of transistors in accordance with Moore’s Law [1]. In recent years, the drive to enhance integrated circuits’ performance by scaling down transistor dimensions has been met with challenges arising from physical limitations and interconnect bottlenecks [2,3]. Thus, the concept of More than Moore has been proposed. One of the key parts of More than Moore is that the improvement of integration capability can be achieved through advanced packaging technology rather than only relying on scaling down transistor dimensions. Different advanced packaging technologies, such as chip stacking, wafer stacking, and through-silicon vias (TSVs), enable the heterogeneous integration of chips with different technology nodes in the same package. For heterogeneous integration, 3D ICs are considered one of the most promising technologies for the future, while traditional packaging technology is facing challenges such as long wiring, high power consumption, and large form factor [4,5]. In addition, 3D ICs can provide higher integration density, lower power consumption, and low RC delay [6,7].

3D ICs can be realized using TSV interconnect technology to achieve vertical stacking structures, resulting in higher integration density and improved performance. Hybrid bonding is a key technology for 3D ICs, enabling fine-pitch bonding of different materials such as Cu/dielectric and Cu/polymer. However, the high thermal budget of the bonding process caused by Cu oxidation poses a challenge for Cu-Cu bonding. The high bonding temperature requirement can result in wafer warpage, bonding misalignment, and compatibility issues with the back-end-of-line process. To overcome this, low-temperature Cu-Cu bonding and low-temperature hybrid bonding for Cu/SiO_2_, Cu/SiCN, and Cu/polymer hybrid bonding have been developed. This paper presents an extensive survey of techniques to achieve low-temperature Cu-Cu bonding and low-temperature hybrid bonding. Moreover, this paper introduces a novel hybrid bonding scheme for metal/polymer interfaces that achieves good flatness and an excellent bonding interface without the need for the CMP process. Low-temperature hybrid bonding can achieve reliable connections between different materials with different coefficients of thermal expansion (CTEs) and can increase the bonding strength.

In this paper, two bonding technologies will be introduced: thermal compression bonding (TCB) and room-temperature bonding with post-annealing. TCB involves applying pressure and raising the temperature during the bonding process. It offers the advantage of more tolerance for surface roughness but has a lower throughput. On the other hand, room-temperature bonding with post-annealing requires lower surface roughness and highly hydrophilic surfaces but allows for batch annealing after bonding, leading to significantly higher throughput. Furthermore, owing to its ability to achieve highly accurate alignment through its spontaneous bonding properties, this technique has established itself as a major bonding technique in advanced packaging [8]. In light of the above, room-temperature bonding with post-annealing technology has been extensively adopted in the industry due to these advantages.

## 2. Low-Temperature Cu-Cu Bonding Schemes

The Cu-Cu TCB process refers to a bonding process that utilizes temperature and pressure to facilitate the interdiffusion of copper at the bonding interface. However, the implementation of Cu-Cu TCB presents several challenges. One major issue is the formation of surface oxide layers in an atmospheric environment, which can impede the interdiffusion of Cu atoms and negatively impact the quality of the bond. Therefore, conventional Cu-Cu bonding requires a high bonding temperature of about 400 °C for the Cu interdiffusion after the TCB process [9,10]. High bonding temperature for the TCB process induces a high thermal budget issue, which may damage IC devices. Thus, it is very important to investigate approaches that can reduce the bonding temperature in Cu-Cu bonding. From the past research results, Cu-Cu bonding temperature can be reduced through surface pretreatment, surface activation, structure modification, orientation control, etc.

For Cu-Cu bonding quality, maintaining a pristine Cu surface is necessary. However, Cu is prone to oxidation, transforming the surface into Cu_2_O [11]. If there were no residual pure Cu atoms within the Cu_2_O layer, further oxidation would still occur under higher temperature conditions, culminating in the formation of CuO [12,13]. This oxidation significantly increases the resistivity of the Cu film. To counteract this, Cu surfaces are typically cleaned to eliminate Cu oxides before bonding. Various techniques, such as in situ cleaning or controlling the interval between cleaning and bonding, are employed to minimize Cu oxide presence. It has been reported that residual Cu oxides can dissolve into Cu during vacuum annealing [14]. For partially oxidized Cu films, theCu_2_O concentration starts to diminish at annealing temperatures between 200 °C and 300 °C. In contrast, fully oxidized Cu films necessitate higher temperatures for Cu_2_O dissolution. Therefore, the aforementioned techniques can be used to reduce the amount of surface Cu oxides to lower the required process temperature for Cu-Cu bonding.

### 2.1. Surface Pretreatment for Cu-Cu Bonding

**Figure 1:**
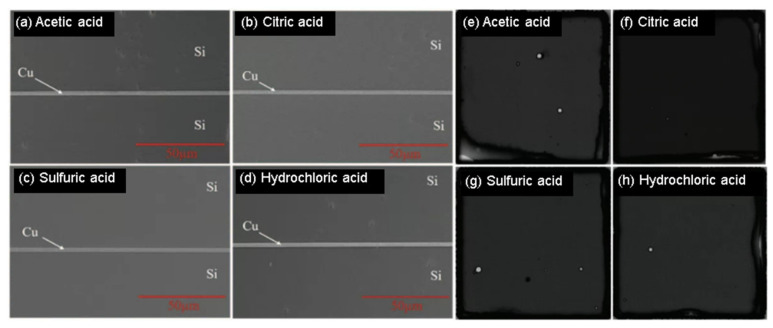
Cross-sectional view of SEM image with (**a**) acetic acid, (**b**) citric acid, (**c**) sulfuric acid, and (**d**) hydro-chloric acid and SAT analysis with (**e**) acetic acid, (**f**) citric acid, (**g**) sulfuric acid, and (**h**) hydrochloric acid pretreatment for chip level Cu-Cu bonding [19].

**Figure 2:**
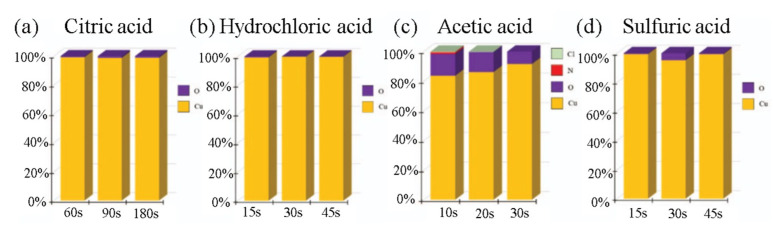
The element component composition at the Cu surface with (**a**) citric acid, (**b**) hydrochloric acid, (**c**) acetic acid, and (**d**) sulfuric acid pretreatment [19].

Cu-Cu bonding has emerged as a promising technique for 3D integration due to its good thermal and electrical conductivity. However, the Cu surface is easily oxidized and contaminated during the manufacturing process, leading to poor bonding quality. To improve the bonding performance, various chemical pretreatments have been investigated to lower the Cu-Cu bonding temperature by removing surface oxides [15,16,17,18]. K.-N. Chen’s group has analyzed and compared the effects of some acids on Cu-Cu bonding through the TCB process; the pretreatments include citric acid, hydrochloric acid, acetic acid, and sulfuric acid [19]. Figure 1 shows the cross-sectional view of scanning electron microscope (SEM) images and scanning acoustic tomography (SAT) analysis of chip-level Cu-Cu bonding with different chemical treatments. As shown in Figure 2, after chemical treatment and plasma treatment, the oxygen content and bonding temperature can be effectively reduced. In this demonstration, the Cu interface was immersed in a chemical acid to remove the oxide layer on the Cu surface, further reducing the bonding temperature to 250 °C in the atmosphere.

### 2.2. Surface Activation Bonding Scheme

**Figure 3:**
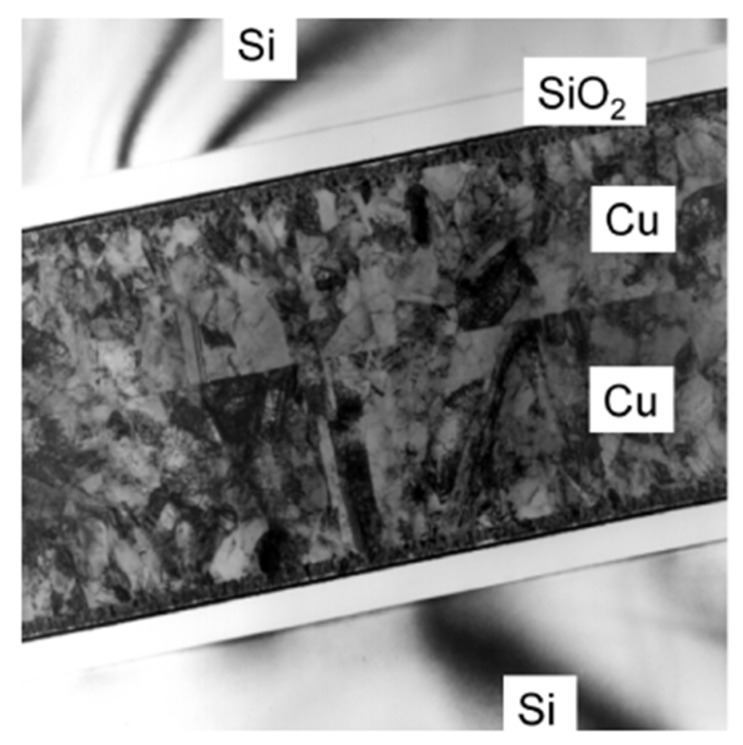
Cu-Cu bonded at room temperature using SAB method [23].

In the surface activation bonding (SAB) process within an ultra-high vacuum (UHV) environment, ions or fast atom beams are utilized to activate the bonding surface. This activation step enables the formation of chemical bonds between the copper surfaces, facilitating Cu-Cu bonding at room temperature. Suga’s group has successfully achieved an ultrafine-pitch bumpless interconnect by utilizing SAB for Cu-Cu direct bonding at room temperature [20,21,22]. Figure 3 shows transmission electron microscope (TEM) images of the Cu-Cu bonding interface prepared by using the SAB method at room temperature [23,24]. Different from traditional Cu-Cu direct bonding, the bonding interface produced using the SAB method had no significant diffusion or growth of Cu grains across the bonding interface. This suggests that the SAB method is not strongly dependent on Cu diffusion and the microstructure of Cu grains. The SAB method is advantageous in terms of avoiding thermal-related issues such as thermal stress, thermal expansion, and expansion-induced bonding misalignment. Furthermore, they have demonstrated the high potential and applicability of this technology by implementing the Cu bumpless structure for interconnecting a thinned flash memory chip and an interposer. The room temperature bonding and good bonding strength are advantageous for the SAB technique. In addition, with the advancement of SAB tools, there has also been a significant increase in throughput.

### 2.3. Structure Modification with Passivation Scheme

Cu-Cu bonding through the passivation scheme is a promising technique in 3D IC integration due to its potential for high-density interconnects with low bonding temperatures and pressures. In this method, the bonding surface of Cu is first passivated by a thin layer of passivation metal material, which reduces the formation of Cu oxide and enhances the bonding strength of the Cu surface [25,26,27].

**Figure 4:**
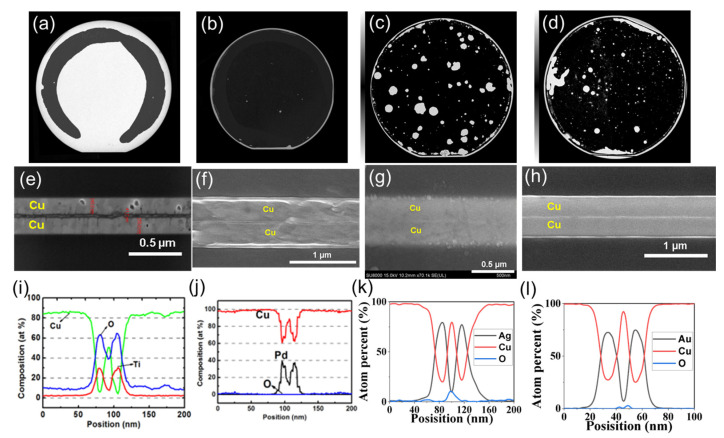
SAT analysis of wafer-to-wafer bonding with passivation schemes (**a**) Ti, (**b**) Pd, (**c**) Ag, and (**d**) Au; cross-sectional view of SEM image for (**e**) Ti, (**f**) Pd, (**g**) Ag, and (**h**) Au; and bonding interface EDX line scan analysis for (**i**) Ti, (**j**) Pd, (**k**) Ag, and (**l**) Au [28,29,30,31,32,33,34].

**Figure 5:**
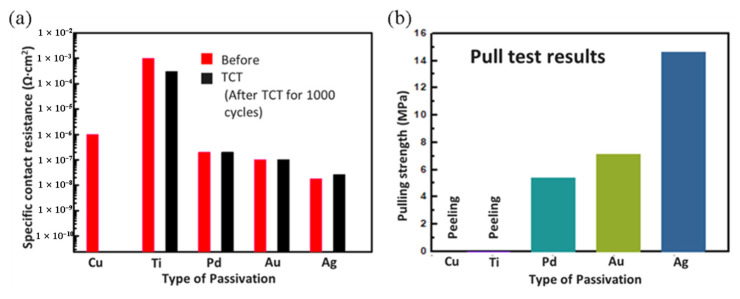
(**a**) Before and after TCT of electrical properties. (**b**) Bonding strength result comparison of without passivation layer and with different passivation layers [28,29,30,31,32,33,34].

Low-temperature Cu-Cu with passivation bonding has been investigated by the K.-N. Chen group, and the passivation metals Pd, Ag, and Au were selected as passivation candidates. Ti was also investigated for its low cost and high compatibility in the semiconductor industry, as shown in Figure 4 [28,29,30,31,32,33,34]. In Figure 4a,e,i, Ti passivation in Cu-Cu bonding was bonded at 180 °C for 50 min. The surface of Ti has a tendency to form a dense oxide layer, which can lead to poor bonding results with significant residual TiOx remaining after the bonding process. In Figure 4b,f,j, Pd passivation in Cu-Cu bonding was bonded at 150 °C for 50 min. In Figure 4c,g,k, Ag passivation in Cu-Cu bonding was bonded at 150 °C for 50 min. In Figure 4d,h,l, Au passivation in Cu-Cu bonding was bonded at 100–150 °C for 30–50 min, and these sample were bonded in a 10^−5^ torr environment through the TCB process. Pd, Ag, and Au passivation could all effectively prevent Cu oxidation and provide successful bonding at 150 °C. In Figure 4f,g,h, good bonding interfaces without any void can be observed. Figure 5a,b show the electrical properties before and after the thermal cycling test (TCT) and bonding strength results [28,29,30,31,32,33,34]. Considering both electrical properties and bonding strength, Au and Ag passivation are the most viable options for low-temperature Cu-Cu bonding.

### 2.4. Orientation Control with (111) Surface of Nanotwinned-Cu

The twin boundary orientation plays a critical role in determining the bonding strength and electrical properties of nanotwinned-Cu (nt-Cu) [35]. Specifically, the twin boundaries with the (111) orientation are expected to provide the most desirable mechanical and electrical properties due to their high density, coherent nature, and specific crystallographic orientation [36]. Therefore, controlling the orientation of nt-Cu with the (111) twin boundaries is crucial for achieving high-performance Cu-Cu bonding.

**Figure 6:**
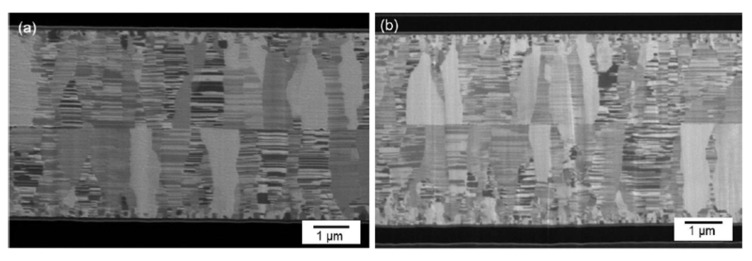
Cross-sectional FIB image of the typical CMP nt-Cu films bonded at (**a**) 150 °C for 60 min and (**b**) 200 °C for 5 min [40].

**Figure 7:**
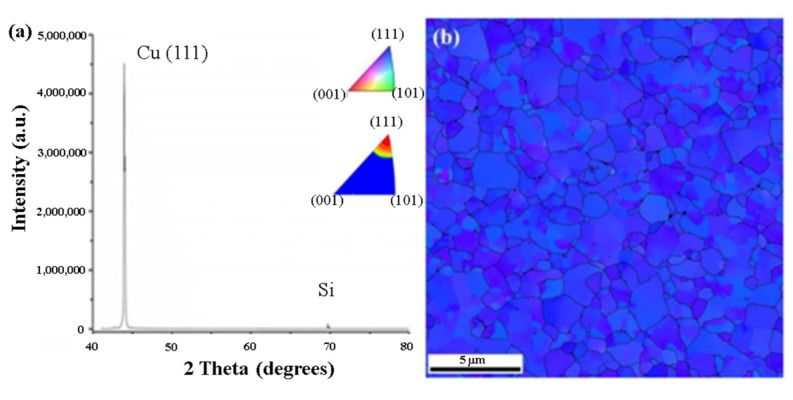
(**a**) XRD pattern and (**b**) plane-view EBSD image of the electroplated nt-Cu films [40].

**Figure 8:**
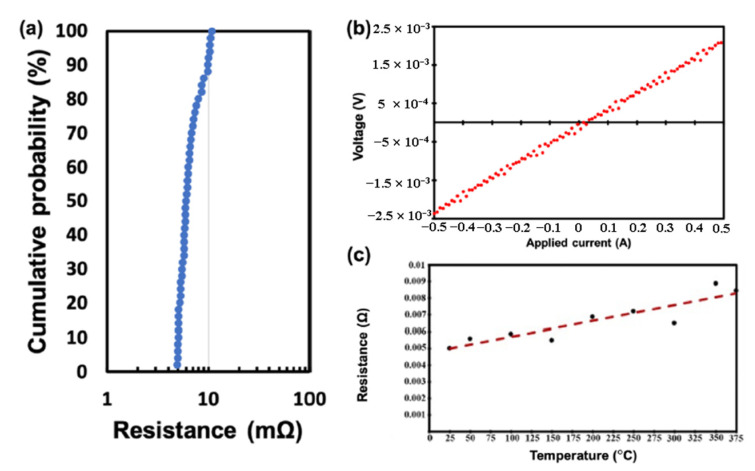
(**a**) Measured cumulative resistance for single Cu-Cu joint from four-point probes; (**b**) I–V curves; (**c**) resistance against measured temperatures from 25 °C to 375 °C [37].

It has been reported that Cu surface diffusion on the (111) surface is the most beneficial for Cu-Cu bonding, which can be achieved at a bonding temperature of 150 °C [37,38,39]. Several methods have been proposed to control the orientation of nt-Cu with the (111) twin boundaries. One of the effective methods is CMP with an appropriate slurry. CMP can selectively remove the (100) grains and preferentially expose the (111) grains in the surface layer of nt-Cu [40,41]. Figure 6a,b show the cross-sectional view of the FIB image after the CMP process and bonding at 150 °C for 60 min and 200 °C for 5 min through the TCB process [40]. In Figure 7a,b, the plan-view orientation image map obtained through electron backscatter diffraction (EBSD) and the X-ray diffraction (XRD) pattern presented provide evidence of the significant improvement in the (111) orientation of the surface [40]. The nt-Cu and SiO_2_ hybrid bond have outstanding electrical properties even at a low bonding temperature of 200 °C. Four-point probe measurements of electrical resistance were performed after the post-annealing process. A Kelvin structure was used in the wafer-to-wafer hybrid bonding approach, and 50 contact structures were measured. The average resistance of 50 joints was calculated to be 1.5 mΩ, resulting in a specific contact resistance of 1.2 × 10^9^ Ω-cm^2^, as shown in Figure 8a [37]. The average resistance was 6.7 mΩ, with slight variations of ±1.75 mΩ. Linear I–V curves were obtained with currents ranging from −0.5 A to 0.5 A, as shown in Figure 8b. The resistance was also measured as a function of temperature up to 375 °C, as shown in Figure 8c. The results indicate excellent thermal stability within the hybrid bonds. The (111) grains exposed by the surface treatment have a smooth and flat surface with a low surface roughness, which is beneficial for achieving a high-quality bonding interface. The bonding temperature, advantages, and disadvantages of the aforementioned Cu-Cu bonding techniques are compared in Table 1.

## 3. Low-Temperature Hybrid Bonding Schemes

**Figure 9:**
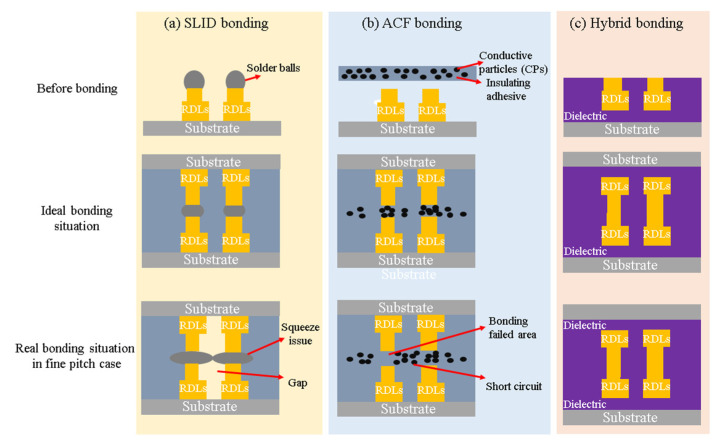
Hybrid structure for three different bonding methods: (**a**) SLID bonding, (**b**) ACF bonding, (**c**) hybrid bonding.

As mentioned above, low-temperature Cu-Cu bonding at temperatures lower than 200 °C can be achieved using these technologies. However, there are still oxidation and reliability issues in pure Cu-Cu bonding. Therefore, it is important to protect the bonded metal from oxidation when a hybrid structure with metal and dielectric materials is used. Figure 9 shows different hybrid structure schemes. Hybrid structures like solid–liquid interdiffusion (SLID) bonding with underfill and anisotropic conductive film (ACF/ACP) materials have been commonly used in the industry. Nevertheless, these technologies cannot meet the needs of the next-generation chip package interconnection technology. SLID bonding squeezes out the solder bump and has a narrow gap issue, so SLID bonding limits the fundamental scaling between two bonding solder bumps. ACF material is composed of conductive particles (CPs) and insulating adhesives, which may cause short circuit issues. Thus, achieving the fine-pitch process in the next generation is an imminent requirement. Hybrid bonding is considered the most promising technology to realize fine-pitch interconnection. In recent research, hybrid bonding technology has been used to achieve fine-pitch interconnection within 10 μm. Currently, hybrid bonding can be divided into metal/dielectric-based (metal/SiO_2_, metal/SiCN, and metal/polymer) structures.

### 3.1. Cu/SiO_2_ Hybrid Bonding

**Figure 10:**
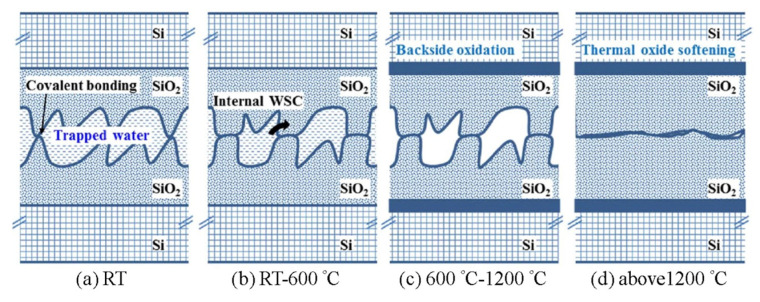
Schematic view of the hydrophilic chemical SiO_2_/SiO_2_ bonding mechanism from room temperature to 1200 °C [42].

Dielectric material is commonly used in the semiconductor industry for standard fabrication processes. Compared to oxide-based and polymer-based hybrid bonding, oxide-based hybrid bonding has a higher requirement of surface cleanliness and surface roughness because its material property is more rigid than that of polymer. Figure 10a–d show the SiO_2_/SiO_2_ bonding mechanism [42]. Following initial bonding at room temperature, covalent bonds form at the roughness contact points. These bonds contribute to the attraction between wafers, aiming to optimize the adhesive area, subsequently inducing internal stress. During the annealing process from room temperature to 600 °C, trapped water is allowed to permeate the stressed oxide due to water stress corrosion (WSC), possibly facilitating the softening of the rough surface. This process might encourage further covalent bond formation, amplifying the bonding energy.

Furthermore, after the plasma activation process, the surface becomes more hydrophilic, and the volume of trapped water at the bonding interface increases [42,43]. This increased water presence, in tandem with the compromised mechanical attributes of the plasma-treated subsurface, likely promotes higher bonding energy. This additional water might be viewed as an adhesive enhancer in the direct bonding mechanism. Furthermore, internal WSC is instrumental in interpreting the augmentation of direct bonding energy, playing a pivotal role in the mechanical progression of the bonding interface.

**Figure 11:**
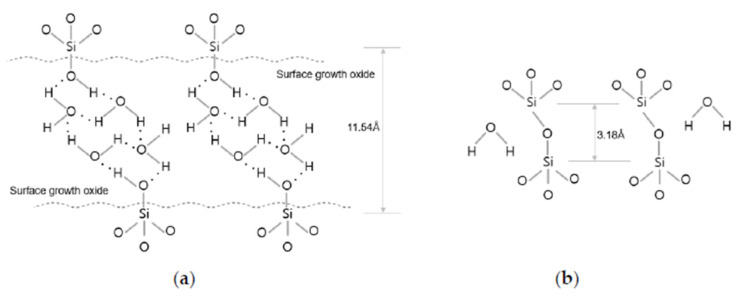
Description of SiO_2_ bonding mechanism. (**a**) After the bonding process at room temperature. (**b**) After annealing process (≥150 °C) [44].

The typical Cu/SiO_2_ hybrid bonding process includes the following: (1) using O_2_/N_2_/Ar plasma activation to promote the dangling bonds and interactions among hydroxyl groups and water molecules; (2) defect removal via deionized water cleansing and scrubbing. Subsequent bonding relies on van der Waals forces and hydrogen bonds between a few water monolayers of water molecules and polar OH groups, which terminate at both the native and thermal SiO_2_; (3) the interface’s van der Waals connections between H_2_O molecules evolve. The annealing process removes the interface water and the covalent bonds are formed, as depicted in Figure 11 [44].

**Figure 12:**
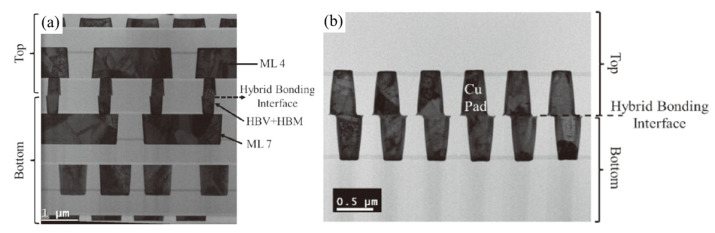
TEM cross-sectional images: (**a**) a daisy chain structure with Cu pads measuring 300 nm in width and the Cu/SiO_2_ hybrid bonding demonstration. (**b**) A successfully bonded structure with 300 nm wide Cu HBM pads [48].

The room-temperature bonding with post-annealing technology uses the spontaneous adhesion of two wafers’ contact at room temperature for a short bonding time. To achieve a good-quality Cu/SiO_2_ hybrid bonding structure, stringent environmental conditions and flat surfaces are necessary, and the critical root mean square (RMS) roughness should be less than 0.5 nm [45,46]. Figure 12 shows the TEM cross-section images for the fine-pitch Cu/SiO_2_ hybrid bonding structure [47,48]. After room temperature wafer-to-wafer hybrid bonding, the post-annealing process was performed at 380 °C for 2 h. The TEM images show the void-free bonding interface and excellent alignment accuracy that can be achieved.

**Figure 13:**
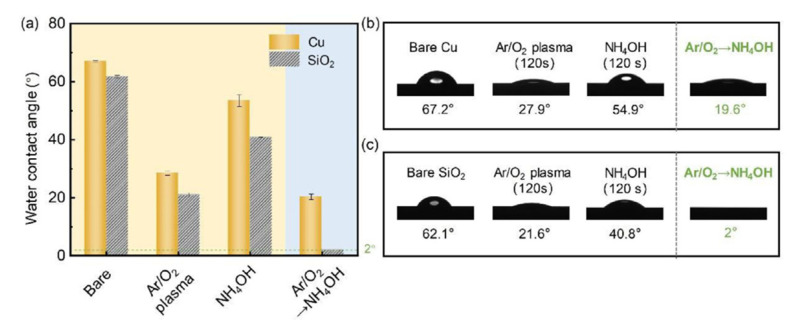
Wettability characterization of the Cu/SiO_2_ hybrid surface. (**a**) Water contact angle of Cu and SiO_2_ before and after different activations. The corresponding contact angle images in (**a**) are shown in (**b**,**c**) [50].

**Figure 14:**
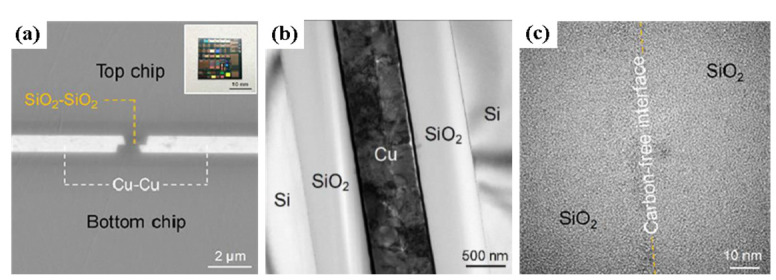
Cross-section of Cu/SiO_2_ hybrid bonding obtained using Ar/O_2_→NH_4_OH schemes. (**a**) SEM cross-section image of Cu/SiO_2_ hybrid bonding interface and its hybrid sample inserted. (**b**) TEM image of Cu-Cu bonding interface. (**c**) Cross-sectional TEM image of the SiO_2_–SiO_2_ interface [50].

Based on Section 2.1, low-temperature Cu/SiO_2_ with surface activation has been investigated by C. Wang’s group [49,50]. As shown in Figure 11, the original Cu and SiO_2_ surfaces demonstrated hydrophobicity due to organic impurities. However, after Ar/O_2_ plasma treatment, a noticeable enhancement in the chemical affinity for both Cu and SiO_2_ emerged, signifying hydrophilic conversion. Remarkably, Ar/O_2_ plasma followed by NH_4_OH causes a pronounced drop in the water contact angle (CA) for Cu (CA = 19.6°) and SiO_2_ (CA < 2°), showing effective co-hydrophilization of the hybrid surface. The bonding result of chip-to-chip Cu/SiO_2_ hybrid bonding with surface activation is shown in Figure 13. Cu/SiO_2_ hybrid bonding through TCB technology with the surface roughness R_a_ below 0.2 nm, involving bonding at 200 °C for 30 min and post-annealing at 200 °C for 2 h, demonstrated exceptional bonding, quality as shown in Figure 14.

**Figure 15:**
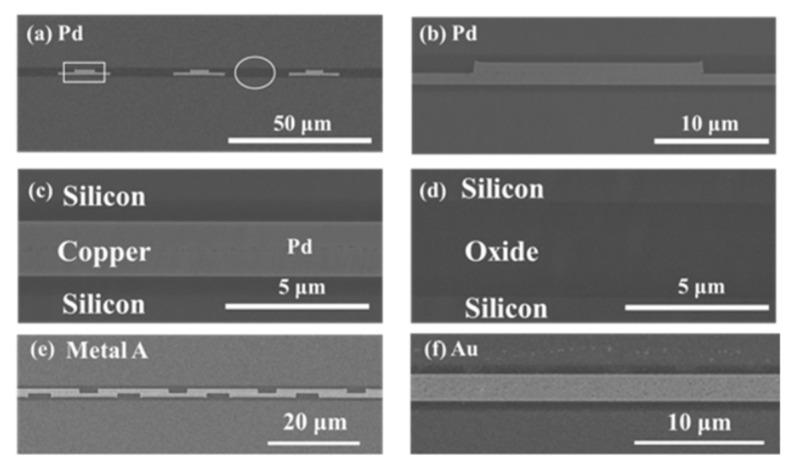
Cu/SiO_2_ hybrid bonding with passivation schemes (**a**–**d**) Pd, (**e**) metal A, and (**f**) Au [52].

Another case of the TCB process is based on Section 2.3; low-temperature Cu/SiO_2_ with passivation structure has been investigated by K. -N. Chen’s group [51,52]. This investigation is necessary for the heterogeneous integration and advanced package application, which can effectively reduce the thermal budget, increase thermal and electrical reliability and increase I/O counts. Figure 15a–f show the chip-to-chip Cu/SiO_2_ hybrid bonding with Pd passivation, metal A passivation, and Au passivation. The Cu/SiO_2_ hybrid bonding through TCB with the surface roughness Ra below 3 nm, bonded at 120–150 °C, demonstrated excellent bonding quality [52]. As mentioned above, the metal passivation scheme can significantly reduce bonding temperature and has been applied for chip-to-chip Cu/SiO_2_ thermal compressive hybrid bonding at 120–150 °C for 1 min in the atmosphere. Compared with chip-to-chip Cu/SiO_2_ hybrid bonding without a passivation scheme, the bonding temperature needs to be more than 250 °C for 5 min.

### 3.2. Cu/SiCN Hybrid Bonding

**Figure 16:**
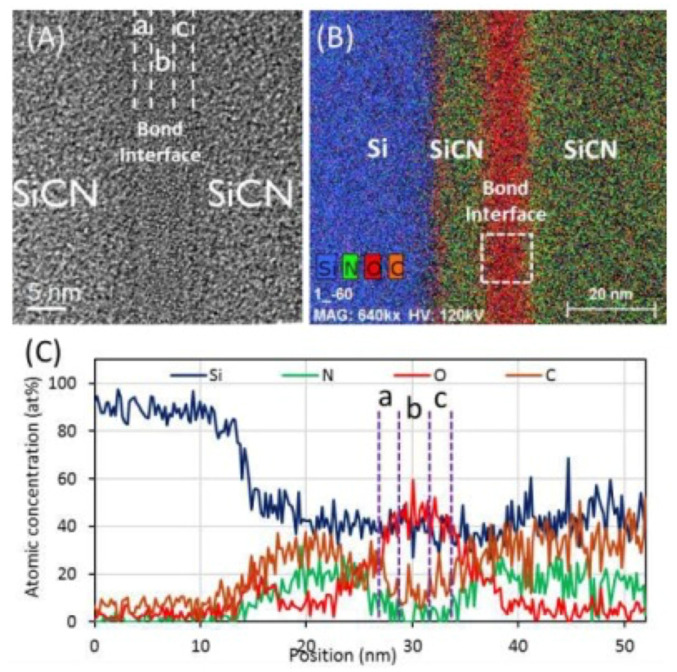
(**A**) SiCN-SiCN bonding interface inspection through TEM analysis. (**B**) EDS colored raw map of SiCN-SiCN at bonding interface. (**C**) The atomic concentration of Si, N, O, and C across the layer stack; region a to region c correspond to bonding interface of (**A**) [53].

Similar to the material properties of SiO_2_, because of its characteristic of being hard and non-deformed, the surface roughness and flatness should be less than enough to ensure void-free bonding. In this section, we will show the SiCN-SiCN and Cu/SiCN hybrid bonding schemes through TCB and room-temperature bonding with post-annealing. Figure 16A–C show the TEM analysis of the SiCN-SiCN bonding interface [53]. After SiCN-SiCN bonding and the annealing process at 200 °C for 2 h, the multiple interfacial layers at the bonding interface were observed. In Figure 13b,c, we can observe an approximately 10 nm oxide-rich layer through energy dispersive X-ray microanalysis (EDX) analysis. From electron energy loss spectroscopy (EELS) analysis, the atomic concentration has been confirmed. Similar to Figure 13a, the spectra are divided into three regions at the bonding interface. From region a to b and region c to b, the concentration of carbon and nitrogen gradually decreases, the concentration of oxygen gradually increases, and the highest concentration of oxygen is observed in region b.

**Figure 17:**
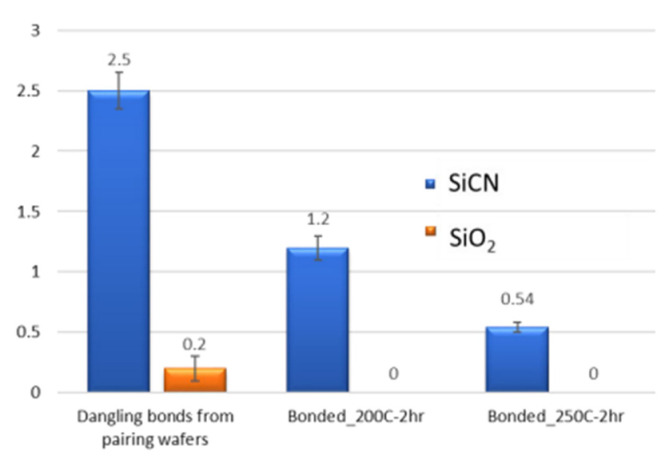
The densities of dangling bonds in SiCN and SiO_2_ before and after bonding with different annealing conditions [53].

Figure 17 presents the inferred dangling bond densities for SiCN and SiO_2_ in different annealing conditions [53]. Electron spin resonance (ESR) monitoring was employed to detect dangling-bond-type defects and to compare the bonding quality between the SiCN-SiCN and SiO_2_-SiO_2_ interfaces. In the initial condition, dangling bond densities are extracted from the surface-activated specimen before bonding. The total dangling bond densities of the paring wafers are estimated to be 2.4 × 10^14^/cm^2^ for SiCN and 0.2 × 10^14^/cm^2^ for SiO_2_ bonding. After bonding, the dangling bond densities of SiCN-SiCN bonded specimens decrease to 1.2 × 10^14^/cm^2^ and 0.54 × 10^14^/cm^2^ after annealing at 200 °C and 250 °C, respectively. In contrast, the minimal dangling bond is present for SiO_2_-SiO_2_ after annealing, indicating that the effect of dangling bonds does not make a significant contribution to chemical bond formation at the interface of SiO_2_-SiO_2_ [53,54]. It is important that subjecting the unbonded SiCN specimens to a 200 °C annealing resulted in minimal change in the dangling bond density. Therefore, it can be inferred that the significant reduction in dangling bonds during the bonding process and subsequent annealing primarily occurred at the SiCN-SiCN interface, which contributed to the chemical reaction and improved the bonding strength.

**Figure 18:**
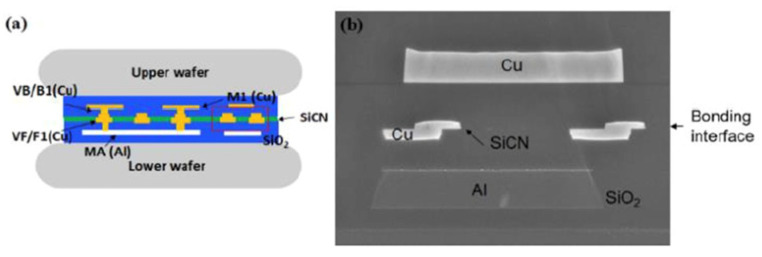
(**a**) Schematic of top and bottom wafer bonding structure and (**b**) SEM cross-sectional image of a well-bonded sample. The observation of the Cu/SiCN hybrid bonding [55].

Based on Section 2.4, the bonding result of wafer-to-wafer Cu/SiCN hybrid bonding with the (111) surface has been performed. The Cu/SiCN hybrid bonding through TCB technology with the surface roughness Ra below 0.44 nm, bonded at 200 °C for 1 h with bonding force of 75 kN, demonstrated excellent bonding quality, as shown in Figure 18 [55]. Furthermore, after the reliability test, good bonding strength can be obtained. The pull test results after 0, 250, 500, and 1000 cycles TCT correspond to 69.1, 68.6, 53.5, and 58.5 kgw.

**Figure 19:**
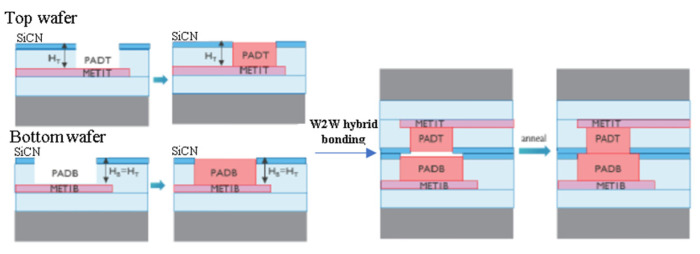
Cross-sectional view of Cu PAD module for the wafer-to-wafer bonding [56].

**Figure 20:**
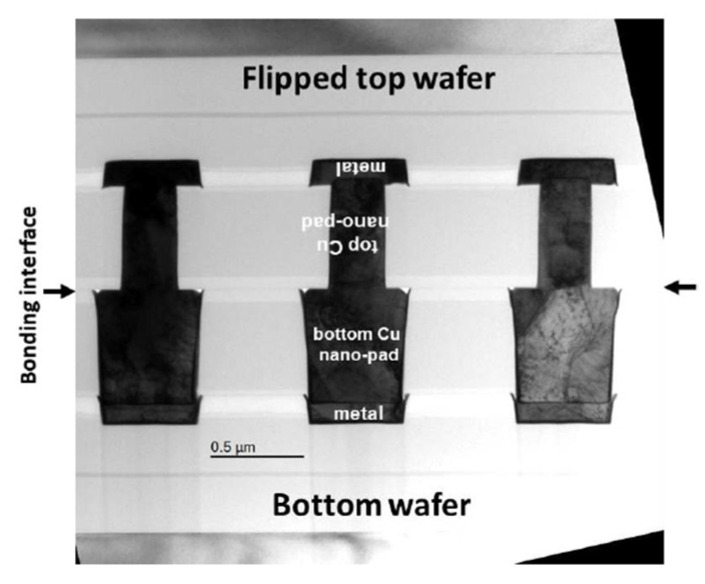
TEM observation at the Cu/SiCN to Cu/SiCN hybrid bonding interface [58].

In recent years, Cu/SiCN interconnection technology by room-temperature bonding with post-annealing has been researched by the Interuniversity Microelectronics Centre (imec) to realize fine-pitch Cu/SiCN hybrid bonding [56,57,58]. In the cross-sectional view of the Cu/SiCN hybrid bonding structure, as shown in Figure 19, the top wafer and bottom wafer of the Cu pad are tuned to control the pad height in the CMP process for electrical performance optimization. In this research, to ensure good bonding quality, the prebonding SiCN surface roughness should be lower than 0.15 nm, and the profile/slope of SiCN needs to be smaller than 1 nm/µm after the CMP process. The other key point of the Cu/SiCN hybrid bonding structure is unequal pad size; the discrepancy in pad size between two wafers can be compensated for during the bonding process due to its ability to counterbalance the alignment tolerance. To achieve vertical connections between two unequal Cu nano-pads, a good surface control procedure is required to precisely construct the opposite profile of the Cu pad to the SiCN dielectric field. Furthermore, wafers with double thickness were annealed through a two-step process to achieve good bonding quality. After room temperature bonding, the wafers were heated to 250 °C to enhance the adhesion strength of the brittle SiCN-SiCN bonding interface. Figure 20 shows the cross-sectional view of TEM analysis. For this hybrid bonding structure, we can observe three bonding interfaces, SiCN-SiCN between each Cu nano-pad, Cu-Cu, and SiCN-Cu, and all these interfaces are free from voids [58].

### 3.3. Cu/Polymer Hybrid Bonding

In recent years, in addition to the Cu/SiO_2_ and Cu/SiCN hybrid bonding mentioned above, Cu/polymer hybrid bonding has attracted significant attention as a promising candidate approach in the industry. Notably, unlike the SiO_2_-SiO_2_ bonding mechanism where van der Waals forces assist in dielectric bonding, the primary bonding mechanism in polymer–polymer bonding is due to the crosslinking of polymers through the heating process. Hence, the primary technology for Cu/polymer hybrid bonding is currently the TCB. Furthermore, for Cu/SiO_2_ and Cu/SiCN hybrid bonding, surface roughness and flatness should be enough to ensure void-free bonding. In comparison, for Cu/polymer bonding, surface roughness and flatness have a higher tolerance to achieve a good bonding interface. Furthermore, the favorable film properties of organic dielectric materials have been widely used in the industry. For example, due to their high elongation capacity, organic materials can effectively absorb the package stress resulting from internal stress [59,60]. However, compared with SiO_2_, the CTE is about 0.55–0.75 ppm/K, the CTE of the polymer is usually higher than 50 ppm/K, and the CTE of Cu is about 17 ppm/K, so we can observe that the CTE mismatch of Cu/polymer is more serious than Cu/SiO_2_ hybrid bonding. Table 2 shows the comparison of these three dielectrics in the hybrid bonding application.

**Figure 21:**
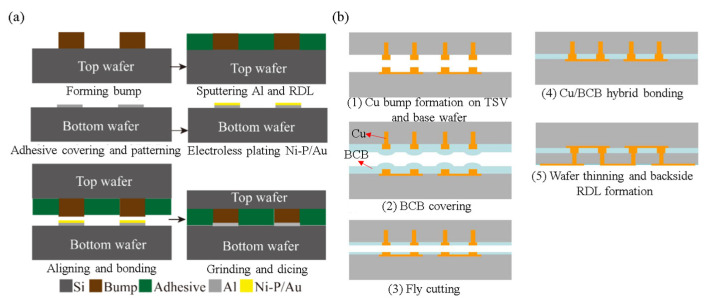
Process flow of (**a**) the asymmetry Cu/polymer hybrid bonding and (**b**) the symmetry Cu/polymer hybrid bonding for 3D integration [63,64].

**Figure 22:**
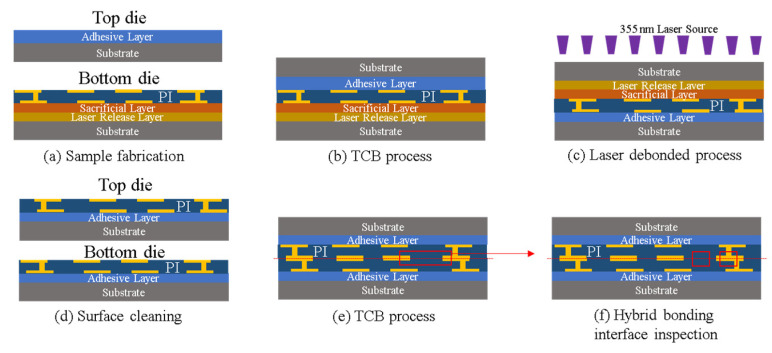
The novel process flow of symmetry metal/polymer hybrid bonding.

**Figure 23:**
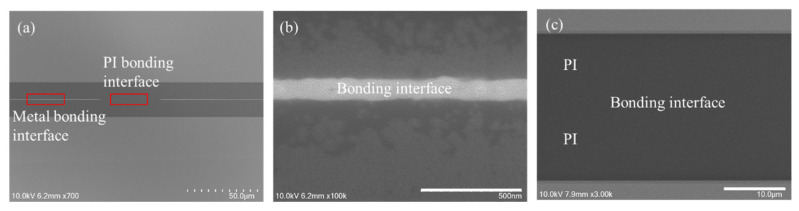
SEM images of (**a**) metal/polymer hybrid bonding interface, (**b**) metal bonding interface, and (**c**) polymer bonding interface.

As shown in Figure 21, for Cu/polymer hybrid bonding, to avoid CMP or fly-cut processes, some researchers use an asymmetrical hybrid bonding structure [61,62,63]. In contrast, a symmetrical hybrid bonding structure typically requires the CMP or fly-cut process to ensure that the double-sided Cu/polymer structure has sufficient flatness and is on the same plane to achieve a good hybrid bonding interface [64]. In this paper, we will present the symmetry metal/polymer hybrid bonding without the CMP or fly-cut process in the novel hybrid bonding scheme, which still has good flatness and an excellent bonding interface for polymer-based hybrid bonding. The process flow is shown in Figure 22. First, a laser release layer and a sacrificial layer were coated on the bottom die through spin coating. Then, the RDL was fabricated on the sacrificial layer. The top die was coated with a layer of adhesive material through spin coating. In the second step, the top die and bottom die were bonded using a TCB process to ensure a good bonding quality. In the third step, the glass substrate was removed through a laser lift-off process with a 355 nm laser. In the fourth step, the residual laser release layer and the sacrificial layer were removed through wet cleaning and O_2_ plasma cleaning. In the fifth step, the top die and bottom die were bonded through the TCB process. Finally, the metal/polymer hybrid bonding interface was observed through SEM analysis, as shown in Figure 23.

**Table 2:** The comparison of Cu/dielectrics in hybrid bonding application.

	Cu/SiO_2_	Cu/SiCN	Cu/Polyimide	Reference
Surface flatness requirement	Surface flatness requirement	Surface flatness requirement	Surface flatness requirement	[53,54]
CMP process	Needed	Needed	Optional	[58,63]
Dielectric constant	3.9–4.5	4.8–4.9	2.8–3.2	[65,66]
CTE (ppm/K)	0.55–0.75	3.0–4.0	Usually higher than 50	[67,68]

**Figure 24:**
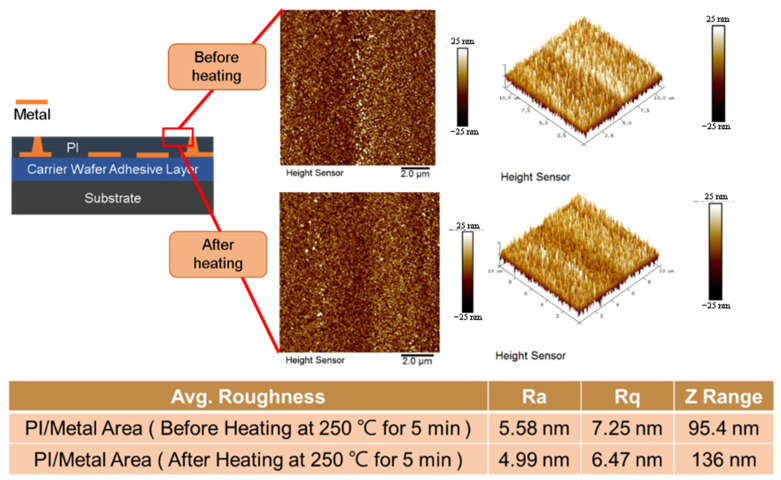
The AFM analysis of metal/polymer hybrid bonding.

In Figure 23, the bonding condition is 180 °C for 5 min without annealing, and a void-free hybrid bond interface can be obtained. To verify the CTE mismatch concern, the higher bonding condition has been simulated (at 250 °C for 5 min). The effect of this temperature on the surface roughness of the metal/polymer interface is shown in Figure 24. The atomic force microscopy (AFM) analysis is shown in Figure 24, which reveals that even when the temperature is raised to 250 °C, the surface roughness of the metal/polymer interface remains significantly unaffected, and good bonding quality still can be obtained at this more severe bonding condition.

## 4. Conclusions

The low-temperature Cu-Cu bonding scheme offers promising solutions for creating reliable and cost-effective interconnects in advanced packaging. The technique involves preventing the formation of a thin layer of Cu oxide from the atmosphere to ensure good bonding quality, such as surface pretreatment, surface activation, structure modification, and orientation control. With its ability to achieve high bonding strength, low resistance, and excellent thermal stability, this technique can be applied to various advanced packaging applications.

On the other hand, the low-temperature hybrid bonding scheme combines the advantages of wafer-level and chip-level bonding techniques, making it a versatile and efficient approach for creating high-density and high-performance 3D interconnects. The technique involves creating Cu-Cu and dielectric–dielectric (SiO_2_/SiCN/polyimide) contact to realize a hybrid structure. In this paper, in addition to introducing these different types of dielectric hybrid bonding, we present a novel hybrid bonding scheme for symmetric metal/polymer bonding that achieves good flatness and an excellent bonding interface without the need for the CMP process. With its ability to achieve fine pitch, high bonding strength, and excellent reliability, this technique offers numerous opportunities for emerging industries such as high-performance computing, data centers, consumer electronics, 5G networks, and artificial intelligence.

## Figures and Tables

**Table 1:** Comparison of different Cu–Cu bonding technologies.

	Bonding Temperature	Advantage	Disadvantage	Reference
Surface chemical pretreatment	250 °C to 350 °C	Low cost High throughput	High bonding temperature	[15,16,17,18,19]
Surface activation	Room temperature	Low thermal budget Good bonding quality (UHV environment)	High cost UHV environment needed	[20,21,22,23,24]
Structure modification with passivation	70 °C to 200 °C	Low bonding temperature Low cost	An additional lithography process for passivation metal deposition	[25,26,27,28,29,30,31,32,33,34]
Orientation control with (111) surface	150 °C to 250 °C	CMOS compatibility Good bonding strength	Limitation in fine-pitch RDL structure	[35,36,37,38,39,40,41]

## Data Availability

The authors can provide the original data upon request, subject to reasonable conditions.
